# Neuropsychology of prefrontal cortex

**DOI:** 10.4103/0019-5545.43634

**Published:** 2008

**Authors:** Shazia Veqar Siddiqui, Ushri Chatterjee, Devvarta Kumar, Aleem Siddiqui, Nishant Goyal

**Affiliations:** Department of Clinical Psychology, Central Institute of Psychiatry, Ranchi, Jharkhand, India

**Keywords:** Attention, executive functions, neuropsychology, prefrontal cortex

## Abstract

The history of clinical frontal lobe study is long and rich which provides valuable insights into neuropsychologic determinants of functions of prefrontal cortex (PFC). PFC is often classified as multimodal association cortex as extremely processed information from various sensory modalities is integrated here in a precise fashion to form the physiologic constructs of memory, perception, and diverse cognitive processes. Human neuropsychologic studies also support the notion of different functional operations within the PFC. The specification of the component ‘executive’ processes and their localization to particular regions of PFC have been implicated in a wide variety of psychiatric disorders.

Human neuropsychology studies brain-behavior relationship, representing a confluence of fields like neurology, psychology, neurophysiology, neurochemistry, and neuropharmacology.[1] The history of clinical frontal lobe study is long and rich. Harlow first presented the case of Phineas Gage who was accidentally struck by a pointed iron bar projected by an explosion which inflicted massive damage to his frontal lobes, apparently destroying the left orbitomedial PFC[2] who went on to develop personality changes. Regrettably, the empirical value of such cases is restricted because of the lesions and insults are not just limited to the frontal region. However, frontal lobe lobectomies as reported by Luria[3] have been informative as they best reveal the characteristic though subtle manifestations of discrete prefrontal damage. On this backdrop, this article tries to fathom the intricacies of prefrontal cortex (PFC) from neuropsychological perspective.

## ANATOMICAL ORGANIZATION OF PFC

### Surface features

Development pattern of frontal lobes involve a hierarchical, dynamic, and multistage process.[4] The anterior part of the frontal lobe referred in the literature as ‘pre’-frontal lobe has been simultaneously referred to as ‘frontal granular cortex’ and ‘frontal association cortex.’ The anterior most portion of the frontal lobe is occupied by the PFC on its medial, lateral, and orbital surfaces. Its relative size reaches a maximum in the human where it constitutes 30% of the cerebral mantle.[5] PFC occupies one-third of the entire human cerebral cortex. The PFC is one of the last cortical regions to undergo full myelination during adolescence in the human.[6] PFC refers to the paralimbic and heteromodal (site for the integration of inputs from more than one sensory modality) components of the frontal lobes. The heteromodal component is known as *granular cortex* whereas the paralimbic component is known as *dysgranular* or *agranular cortex.*[7] This complex and distributed organization reflect the characterization of this brain area as a protean and heterogeneous entity, dedicated to sustaining rapid computations required to accomplish a wide range of mental activities.[8]

## MAJOR SUBDIVISIONS

### Lateral organization

The Broca's area is interposed between the dorsolateral PFC (DLPFC) and the ventral portion of premotor cortex. This region is concerned in the abstract mediation of the verbal expression of language. Occasionally, there are two distinct sulci within the prefrontal region designated as the superior frontal sulcus and inferior frontal sulcus.[8]

### Medial organization

The division between the paracentral and medial frontal gyri is occasionally formed by a vertical sulcus emerging from the cingulate sulcus above the midpoint of the corpus callosum.[9]

### Orbitofrontal organization

The orbitofrontal cortex (OFC) lies in the base of the anterior cranial fossa on the ventral surface of the frontal lobe. It includes area 13 caudally, area 14 medially and cortex on the inferior convexity includes area 12 caudally, and area 11 anteriorly.[10] The posterior portion of the OFC is formed by agranular cortex, and the intermediate portion is formed by dysgranular cortex.[7] This region of cortex is categorized as limbic or paralimbic cortex. In the lateral region of the right dysgranular OFC is a postulated secondary gustatory or taste region[11] Caudal to the secondary gustatory is the orbital extension of primary olfactory cortex.[12] Granular cortex forms the rostral portion of the OFC.

### Connections

Inherent connections of the frontal lobe form vital feed-forward and feedback circuits from the center of prefrontal information processing. The PFC through its extensive association connections is linked with distant and broadly dispersed parts of the association and limbic cortices. Prefrontal interconnections with the amygdala, hypothalamus, midbrain, and pons represent important subcortical linkages of the extended prefrontal neural system. These are likely to integrate higher-order brain functions mediated by the PFC with more developmentally fundamental brain activities such as emotion and visceral, or autonomic, functions.[13] Practically, all-prefrontal connections are reciprocal, exceptional in that regard are the basal ganglia, to which the PFC sends unreciprocated direct efferent.[14] It is also of singular interest that the PFC is the only neocortical region directly projecting to the hypothalamus and the septal region.[15] Different sub-areas of the PFC have different connections. The orbital region is primarily connected to the medial thalamus, hypothalamus, ventromedial caudate, and amygdala; the DLPFC, on the other hand, is primarily connected to the lateral thalamus, the dorsal caudate nucleus the hippocampus, and the neocortex.[16]

## FUNCTIONAL ORGANIZATION OF PFC

PFC is often classified as multimodal association cortex as extremely processed information from various sensory modalities is integrated here in a precise fashion to form the physiologic constructs of memory, perception and intricate action, and diverse cognitive processes are monitored here.[17]

### Lateral prefrontal cortex

Functional magnetic resonance imaging (MRI) and event-related potential (ERP) research have defined the spatial and temporal contributions of lateral prefrontal cortex (LPFC) including portions of inferior, middle, and superior frontal gyri in language, attention, memory,[18] response conflict, novelty processing which is crucial for new learning, creativity, and new learning.[19,20] This region is also responsible for the temporal ordering of events,[21] explicit memory,[22] and metamemory.[23] Simulation, i.e., process of generating internal modes of external reality, the absence of which can lead to stimulus-bound behavior thereby resulting in utilization behavior[24] and reality monitoring is too subserved by this region which is proposed as a major mechanism in self-awareness.[25] The caudal-most portion of the LPFC is important for attention and orientation. DLPFC activation which belongs to a neural circuit that includes posterior parietal cortex, head of the caudate nucleus, and the dorsomedial thalamic nucleus[7] has been associated with a diverse set of cognitive processes, including actively maintaining information in working memory,[26] changing behavior according to task demands[27] or representing past events, current goals, and future predictions[28] and organization and conceptualization of finances.[29] Increased memory-related activity in mid-ventrolateral PFC has been related to actively encoding and retrieving information,[30] updating and maintaining the contents of working memory.[31] Right DLPFC mediates negative attitudes and left ventrolateral PFC mediates positive attitude, spatial and conceptual reasoning process,[32] planning,[9] and integration of perception with action across time.[33]

### Medial PFC

Specifically, the medial frontal region (anterior cingulate area) appears to be involved in bimanual coordination, attention to demanding cognitive tasks, modulation of body arousal, spatial memory, self-initiated movement, and conflict resolution (medial prefrontal and medial orbital regions).[29] The anterior cingulate cortex is also involved in the perception of pain and possibly in mediating the emotional response behind it. Reward and goal-related activity are thought to correspond to the unique patterns of connections that link the rostral cingulate motor cortex with the prefrontal and limbic cortices.[8] Ventromedial region plays a role in decision making[29] and the retrieval of information from long-term memory and metacognitive processes.[34]

### Orbitofrontal cortex

OFC functions as a component of the paralimbic ring involved in autonomic, response inhibition, and stimulus significance functions,[35] mnemonic functions and delayed response.[36] It plays a role in reward expectations[37] and in the anticipation and processing of outcomes even if the outcome does not produce any reward.[38] This region has been shown to have a significant role in social and emotional behavior.[39] Anterior OFC is activated in case of aversive tastes[40] and pleasurable taste is mediated by caudomedial regions of PFC.[41] PFC interactions with the hypothalamus mediate reward aspects of eating like food cravings.[42] Ventral PFC emerging from OFC is connected with limbic system and is involved in emotional processing.[43] This region is intimately associated with amygdala and anterior cingulate, and is involved in behavioral self-regulation.[44]

## THE SOMATIC MARKER HYPOTHESIS: A MODEL FOR SELF-REGULATION

Damasio[35] postulated that the OFC does not contain factual information pertinent to the current contingency, but provides “somatic markers” that enable the individual to “learn by experience.” Damasio postulated that portions of the ventromedial PFC provide a repository for the linkage of current contingencies with the individual's previous emotional experience of similar situations. This linkage of factual sets (held in the appropriate association cortices) and emotional sets (held in the ventromedial frontal cortex) is thought to modify the response of the individual to environmental stimuli and to facilitate logical reasoning. According to this hypothesis, individuals who fail to develop context appropriate somatic markers, either through a “sociopathic temperament” or through injury to the ventromedial frontal cortex will have inappropriate stimulus-bound behavior typical of sociopathy.

## FUNCTIONS OF PFC

### Executive functions

These functions as mediated by the PFC with its rich cortical and subcortical connections include ability to initiate and carry out new and goal-directed patterns of behavior, sustained attention,[3] motor attention, i.e., enactment of action schemas requires attention directed to events in the motor or executive sector[45] short-term memory tasks,[46] inhibitory control of interference, filtering or gating mechanism for information processing[21] working memory,[18] stimulus detection and sequencing tasks,[47] planning, set shifting, flexibility, delayed responding, and active problem solving.[48] Executive functions are closely linked to emotional regulation as well.

### Memory

PFC plays a significant role in encoding and retrieval of memory. Neuroimaging studies found left frontal activation with memory encoding and right PFC activation with retrieval of episodic memory.[49] Studies on frontal lobe patients have yielded important insights on the role of PFC in recent memory,[3] source memory, i.e., memory involving contextual factors associated with learning, sequential memory, i.e., encoding and representation of temporal information.[50]

### Intelligence

Intelligence being a complex construct, certain aspects of it are known to be mediated by PFC. Prominent among these are verbal expression, memory, abstraction, and the ability to formulate behavioral plans and to pursue them to their goal,[51] ability to perceive the spatial relationships between one's self and the environment, or to perform tasks that require the guidance of one's actions by visual information, spatial, or otherwise.[52]

### Language

Neuroimaging, neuropsychologic, and neurophysiologic studies have reported consistently the role PFC in regulating spontaneous speech, narrative expression, and verbal fluency.

### Visual search and gaze control

It involves ability to analyze pictorial detail, and integrative scanning of all the pertinent details.[53] Damage can result in a failure to direct gaze and correct erroneous or unnecessary eye movements in visual tasks under instructions.[3]

## NEUROPSYCHOLOGY OF PFC

### Development and involution

According to Piaget,[54] logical reasoning, which in turn depends upon the cognitive functions of the PFC, does not attain full development till age 12, which is the time when the prefrontal reaches full development. The greater period of development occurs at the age 6-9 years with more moderate effects between ages 9 and 12 years, and performance-moderating adult levels during adolescence and sometimes also until age 20.[55] Further development of frontally mediated executive functions may continue through age 16[56] with continued development throughout adulthood.[57] Working memory develops by age 8 months, which is demonstrated by the successful completion of delayed tasks.[58] By the age of 1.5-5 years, attention, executive and self-reflexive skills emerge.[4] By the age of 5-8 years, cognitive abilities in the area of recognition memory, concept formation, set shifting, and rudimentary planning skills emerge.[59] Both selective attention and exclusionary attention also develop at that time with maximum development at age 6-9 years. Planning and motor memory appear to have the same timetable, possibly also with rapid development between 6 and 9 years.[55]

### Localization *vs.* network model

Do the different regions carry out distinctive functions, e.g., inhibitory control, motor planning, and spatial memory as argued at different times by different investigators[60] or is there a hierarchical relationship between superior and inferior dorsolateral cortex as proposed by Owen and colleagues.[61] In contrast to the popular view, a critical review of functional neuroimaging studies including both positron emission tomography (PET) and functional magnetic resonance imaging (fMRI) studies suggest that specific regions within dorsolateral and ventrolateral PFC make identical functional contributions to both spatial and nonspatial information. Human neuropsychologic studies also support the notion of different functional operations within the PFC.[62] The notion of cortical centers controlling specific capacities has still not entirely disappeared, but it has largely given way to that of networks, i.e., aggregation of interconnected neural loci as the core concept of localization. According to this network model, cognitive functions such as attention, memory, and perception are not localized, but they accompany or emerge from the information processes in the networks. The primary objectives of computational network models of the PFC are to specify certain principles of operation of frontal networks-(i) in the performance of tasks that are known to be sensitive to frontal injury and (ii) in cognitive functions such as short-term memory, planning, inhibitory control that are ascribed to the PFC.[5]

### Are all prefrontal ‘cognitive functions’ ‘executive’ in nature?

The terms ‘executive’ and ‘frontal lobe’ functions are often used interchangeably. The terms should be denoted separately as it cannot be assumed that all of the cognitive functions of the PFC are ‘executive’ in nature. Executive function should not be confounded with prefrontal except at a hypothesis level because of the nonprefrontal contribution to executive function and function of prefrontal lobes that extend beyond the list of cognitive abilities for which executive function is an umbrella[63] [Table 1]

**Table 1:** Important functions and corresponding impairments associated with prefrontal cortex

Function	Impairment
Attention	Impaired attention to novel stimuli, impaired selective attention
Memory and learning	Poor working memory (impairment in brief and transient storage and processing of information)
	Poor temporal memory (difficulty in remembering temporal status of events i.e., difficulty in ascertaining relative recency of different events)
	Poor prospective memory (difficulty in remembering intentions for carrying out future actions)
	Increased sensitivity to proactive interference
Executive functions	Poor planning of behavior
	Poor strategy formation and problem solving
	Poor decision making
	Poor set-shifting
Motor behavior	Unsystematic gaze movements
	Poor movement programming (e.g., problem in alternating movements of hands)
	Poor fine movement
	Motor impersistence, perseveration
	Unilateral hypokinesia and unilateral motor neglect
Speech	Expressive aphasia
	Decreased verbal fluency
Self-control	Grasp reflex (automatic tendency to grip objects coming in contact of hand)
	Groping response (the hand tends to follow and manipulate an object, e.g., holding, rubbing, etc., following tactile stimulation. Apart from hands, often the eyes also follow the object in somewhat magnetic fashion)
	Imitation behavior (tendency to imitate other's gestures or movements)
	Utilization behavior (appropriate movement done in a context that is inappropriate, e.g., while eating the patient may pick up a comb and start combing his hair just because a comb has been kept in front of him by someone)
Abstract thinking	More reliance on concrete and superficial clues
Affect	Inappropriate or blunted affect
Sphincter control	Little or no concern about urinating or defecating at inappropriate places

### Neuropsychologic test findings related to PFC damage

The behavioral changes that occur as a result of damage to PFC are very difficult to capture with many neuropsychologic tests.[50] Patients with large prefrontal lesions can perform within the normal range on tests of memory, intelligence, and other cognitive functions. Even supposedly sensitive tests like Wisconsin Card Sorting Test (WCST) sometimes fail to discriminate patients with frontal lesions from people with normal functions or those with lesions in other regions.[64] Tests such as WCST appear to be more sensitive to dorsolateral than orbitofrontal PFC damage. Other tasks often employed to detect lateral PFC deficits are Stroop test which is particularly sensitive to failures in inhibitory control and tests of divided attention. However, patients with inferior parietal lobe damage also fail tests of divided attention and thus do not provide accurate information. In contrast to lateral PFC, damage to orbitofrontal damage leaves the cognitive skills relatively intact but affects all spheres of social behavior.[65]

## NEUROPSYCHOLOGIC ASSESSMENT OF PREFRONTAL FUNCTION

### Assessment of attention

Most statistical studies have shown that there are two factors that are relevant in our choice of attentional tests. The factors are speed or processing capacity and control or working memory. On this basis, there are three levels of attentional tests.[50]

- Operational level with high speed of information processing where highly structured stimulus driven tasks are used, e.g., Trail Making A test, Stroop Test, and Digit Symbol of WAIS-R.
- Tactical level with tests for focused and/or divided attention where partially structured memory-driven tasks are used, e.g., continuous performance test, paced auditory serial additive tasks.
- Strategic level where unstructured tasks like WCST and Stroop tests are used.

### Assessment of memory

For assessment of short-term memory/working memory, digit span; letter number sequencing (WMS),[66] Ray auditory verbal learning test (RAVLT),[67] California verbal learning test (CVLT)[68] spatial span (WMS), and visual pattern test[69] are used. Battery for assessment of working and logical memory includes Wechsler Memory Scale (WMS-III).[66] Tests for assessment of emotional and executive functioning disorders associated with prefrontal lobe dysfunction are given in Tables 2 and 3, respectively.

**Table 2:** Tests for assessing emotional disorders associated with prefrontal lobe dysfunction

Emotional disorders	Instrument
Anxiety	Hamilton anxiety scale[70]
Depression	Hamilton depression rating scale[71]
Apathy	Apathy evaluation scale[72]
Disruptive behavioral disorders	Overt aggression scale[73]

**Table 3:** Tests for assessing executive functioning associated with prefrontal lobe dysfunction

Tests used	Functions assessed
Wisconsin card sorting test (WCST)[74]	Strategic planning, organized search, learning from feedback
Tower of London[75]	Planning, working memory
Stroop color and word test[76]	Inhibition
Trail making test[77]	Motor speed, shifting set, visual scanning
Behavioral assessment of the dysexecutive syndrome (BADS)[78]	Predicts everyday difficulties
Mazes-WISCIII	Planning
Cambridge neuropsychologic test automated battery (CANTAB)	Planning, working memory
Verbal fluency test[79]	Response test
Cognitive estimates test[80]	Judgment

### Assessment of language functions

Numerous tests of language function have been developed including comprehensive batteries for adults and children as well as “aphasia” screening tests. Some of these include Boston Naming Test[81] which is part of Boston Diagnostic Aphasia Battery, Peabody Picture Vocabulary Test - Revised [PPVT-R] which assesses auditory comprehension of picture names and Token Test[82] which assesses verbal comprehension of commands of increasing severity.

## DISORDERS ASSOCIATED WITH DAMAGE TO PFC

As it has been known that frontal lobe not only mediates cognitive aspects of the personality, but also its affective and emotional aspects as well.

### Apathy

It results from widespread lesions of the PFC. In the affective sphere, the hallmark of the disorder is the generalized blunting of affect and emotional responses. The patient's underlying mood is frequently one of profound indifference, and so is his or her attitude toward others.[16]

### Depression

Experience of depressed mood, can be a result of left PFC lesions, especially those involving anterior (polar) aspects of the frontal lobes.[83]

### Social behavior

PFC lesions are likely to have great impact on social behavior particularly in case of OFC lesion. It commonly results in euphoria. Instinctual urges may be released or exacerbated. Some patients with OFC lesions show a tendency to have voracious appetite, driven to satiate an apparently insatiable hunger. The sexual drive also appears frequently disinhibited by prefrontal, especially orbital lesions.[84]

### Prefrontal syndromes

To have a holistic and comprehensive understanding of these syndromes, a more specific terminology based on structural, functional, and clinical features, which recognize *dorsolateral, medial,* and *orbital* prefrontal regions, was developed.

### Dorsal convexity Dysexecutive syndrome

It is characterized by deficits in cognitive flexibility, temporal ordering of recent events, planning, regulating ones actions based upon internal, and external stimuli.[85] This results in a reduced state of mental control, perseveration, and impairment of sustained attention. The capability to retrieve information is altered despite evidence of intact recognition. Patients present with diminished judgment, impaired working memory, insight, self-care, and there is often a general reduction in verbal and nonverbal fluency. There is impaired priming of stereotypes if the lesion is of ventromedial PFC.[86]

### Medial frontal apathetic syndrome

The hallmark feature of medial apathetic syndrome is a severe reduction in spontaneity, motivation, and lack of interest in the environment. Memory of recent events is relatively intact. It is thought that the overall alteration in motivation and motor activity is a result of the lesion involving the medial motor cortices.[3]

### Orbitofrontal disinhibition syndrome

Patients with OFC damage are characterized generally by an acquired disturbance of personal and social behaviors.[39] There are marked abnormalities in the realms of reasoning, decision-making, and emotional control. This often results in explosive aggressive outbursts characterized by socially unacceptable, tactless, and vulgar presentation.[3]

### Association with expression of psychiatric disorders

The specification of the component “executive” processes and their localization to particular regions have been implicated in a wide variety of psychiatric disorders ranging from depression to anxiety disorders to schizophrenia as well as in a number of other disorders like attention deficit hyperactivity disorder (ADHD), autism, conduct disorder, etc.

#### Schizophrenia:

Findings from WM studies in schizophrenia indicate that schizophrenia patients are consistently impaired on WM tasks irrespective of WM domain or processing requirements. This pattern of WM performance may further implicate DLPFC dysfunction in the liability for schizophrenia and has implications for future cognitive, genetic, and developmental research.

#### Mania:

Study conducted by Lebowitz *et al*. indicated that impairment in verbal fluency was found to be greater with the increase in number of episodes of mania.[87] Both number of episodes and total number of hospitalizations have been found to be related to poorer performance on several aspects of WCST.[88]

#### Depression:

Based on a meta-analysis of 13 studies, Veiel concluded that cognitive deficiencies associated with major depression are similar to those seen in moderately traumatic head injury.[89] Merriam *et al.* reported that unipolar major depression patients demonstrated significant deficits on the WCST.[90]

#### Dementia:

Patients with Alzheimer's disease (AD) and Parkinson's disease can be distinguished on the basis of certain cognitive and behavioral features. Performances on different tests have given rise to the similarities and differences in the cognitive profile of these two groups.[91] Similarities between the groups were seen in visuo-motor speed and attention, but differences were found in executive functioning, memory, sequencing abilities, set shifting, and word fluency. Thus, cortical patients (AD) perform significantly worse than the subcortical group (Parkinson's disease) in the memory abilities while the latter group showed greater deficits on the executive functions. Studies have found that patients with a high-cognitive reserve, i.e., higher education, occupation, etc. attain a higher neuropsychologic performance than those with a low cognitive reserve and this plays an important protective role in the incidence of cognitive deterioration and dementia.[92]

## CONCLUSION

Large-scale distributed networks coordinate all complex behavior domains. The performance of a relevant task engages all components of the pertinent network, and damage to any network component can impair behavior in the relevant domain. Experimental data and lesion based-behavioral analyses and functional imaging observations demonstrate that the appropriate and skilled execution of higher-order tasks depend not only on PFC, but also on the integrity of other cortical and subcortical structures that are interconnected with the PFC.
